# A rare case of radial arteriovenous fistula after transradial coronary intervention

**DOI:** 10.21542/gcsp.2025.32

**Published:** 2025-06-30

**Authors:** Priyadharshini Krishnaswamy, BS Arun, Vinay KS, Shivanand S Patil

**Affiliations:** Sri Jayadeva Institute of Cardiovascular Sciences and Research, Bangalore, India

## Abstract

Radial access for coronary interventions usually has fewer complications than the femoral route. An iatrogenic arteriovenous fistula (AVF) at the access site is a complication sometimes associated with coronary procedures, most often via the transfemoral route. However, it is rarely encountered with transradial access. We report a rare case of iatrogenic right radial arteriovenous fistula in a 52-year-old male following percutaneous transluminal coronary angioplasty (PTCA) via a right radial access. The increasing frequency of right radial access for coronary interventions necessitates awareness and recognition of this potential complication. Furthermore, necessary precautions should be taken to prevent its development.

## Introduction

Coronary interventions- like CAG, PTCA etc., are usually performed via the right radial or femoral arterial routes^1–5^. Radial access is gaining preference over femoral access due to a lower bleeding risk, fewer vascular complications, better patient satisfaction, and reduced morbidity and mortality^1,2,4,5^.

Vascular complications at the access site occur more commonly with femoral access and constitute 1.4% of all PCIs^1^. These include pseudoaneurysms, arteriovenous fistulas (AVF), access site hematomas, retroperitoneal hematomas and dissections^2^. An AVF is an abnormal connection between an artery and a vein with an increased flow across it^7^. Iatrogenic AVF is a rare access site complication seen in 0.86% cardiac catherizations via the femoral access, but only in 0.03% of procedures via the radial access.^2,4^

We report a rare case of right radiocephalic iatrogenic arteriovenous fistula (AVF) in a 52-year-old man following PTCA.

## Case report

A 52-year-old male with a history of hypertension initially presented at a peripheral hospital with acute coronary syndrome-anterior wall ST elevation myocardial infarction and was thrombolysed with intravenous streptokinase. The patient was then referred to our center for further management. The patient had a left ventricular ejection fraction of 43% and normal pulmonary pressure. Coronary angiography performed via the right transradial approach revealed single-vessel disease of the left anterior descending artery (LAD). Successful PTCA with stenting of the LAD was performed via the right transradial approach as a staged procedure 2 days after the angiogram. In both procedures, the puncture was performed without imaging guidance using a 20 G Jelco needle with a single attempt by experienced operators on the team. A 6F radial sheath was used for both procedures. The patient remained stable and asymptomatic during the post-procedure period and was discharged two days later.

A month later, the patient presented to the outpatient department with complaints of persistent dull aching pain at the radial access site with a constant sensation of vibration over the area, often interfering with his sleep for the last two weeks. He did not experience breathlessness, chest pain, palpitations, or edema. On examination, vitals were stable with a heart rate of 75 beats per minute and blood pressure of 120/80 mmHg. Cardiac examination revealed no abnormalities. Local examination of the right radial access site revealed a 1×1 cm swelling with normal overlying skin and a palpable continuous thrill at the site. On auscultation, a continuous bruit was heard over the area.

**Figure 1.**
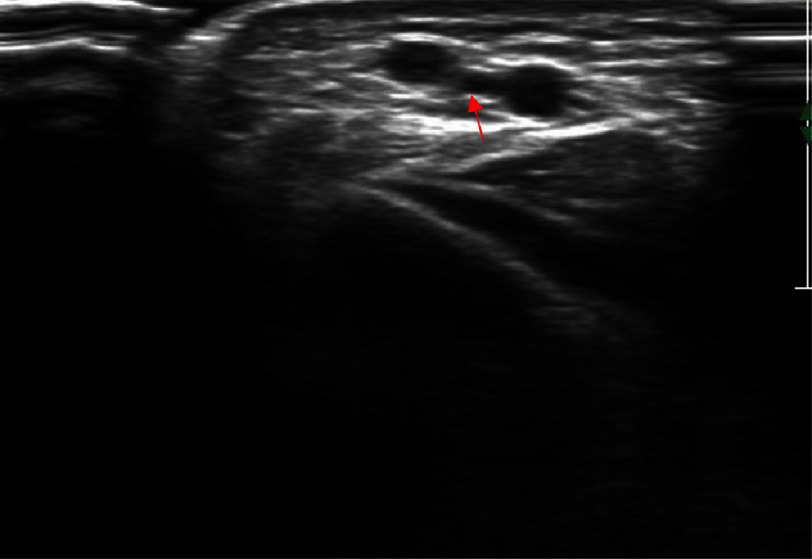
Ultrasound image of the AV fistula, inferior and to the right of the radial artery. The cephalic vein (red arrow) is superior and to the left.

**Figure 2.**
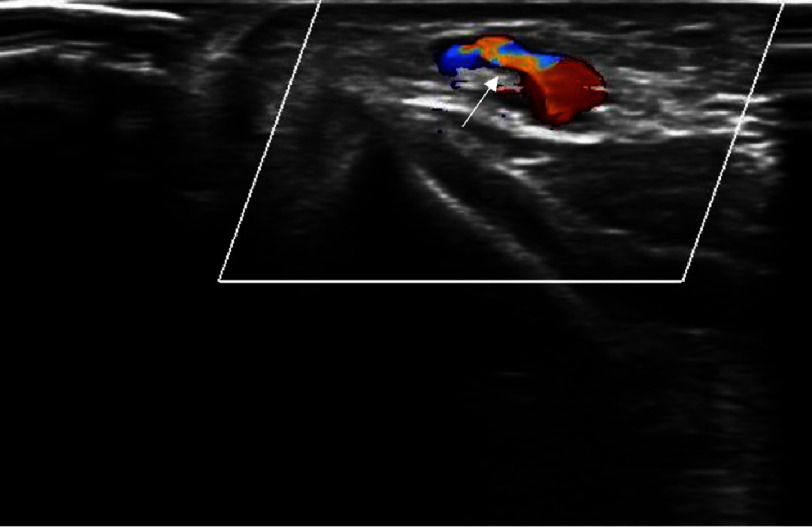
Arterial Doppler showing flow in the AV fistula (white arrow).

**Figure 3.**
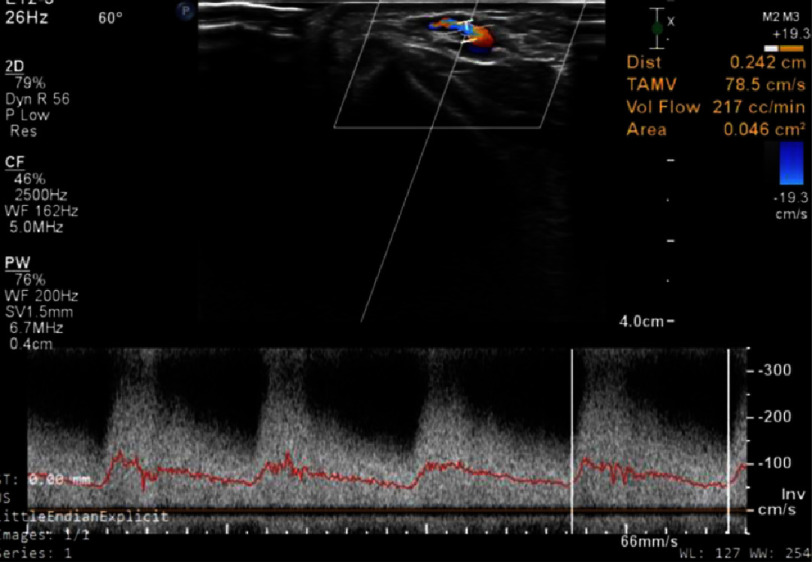
Arterial Doppler showing the flow gradient across the AV fistula.

An arterial Doppler of the right upper limb was performed, which revealed a radiocephalic arteriovenous fistula in the distal forearm, just proximal to the wrist, with a maximum diameter of 2.6 mm and a length of 4 mm. The flow volume across the fistula was 217 cc/min (Figures 1, 2, 3). Vascular surgery was consulted based on these findings to plan further management. A trial of ultrasound-guided compression for three weeks was advised. The neck of the fistula was identified using ultrasound guidance. Under aseptic precautions, with the right arm abducted and externally rotated in the supine position, the ultrasound transducer was placed directly over the neck of the fistula to visualize the artery and vein and their communication.

A sustained pressure was applied with the transducer for 20 min over the neck of the fistula with continuous color Doppler monitoring, such that the flow through the fistula was obliterated while the distal flow in the main radial artery was maintained. On reassessment of the fistula after initial compression, blood flow continued. A compression dressing was applied for 24 h. Compression was repeated after a week in the outpatient clinic. A review of the arterial Doppler of the right upper limb at the end of three weeks revealed no improvement in the size of the fistula or flow volume across it. Therefore, surgical repair was planned, and the patient was admitted for the same. Repeat 2D ECHO showed an ejection fraction of 45% with anterior wall hypokinesia, trivial tricuspid regurgitation with mild pulmonary artery hypertension (pulmonary artery systolic pressure, 38 mmHg), and a collapsing inferior vena cava.

**Figure 4.**
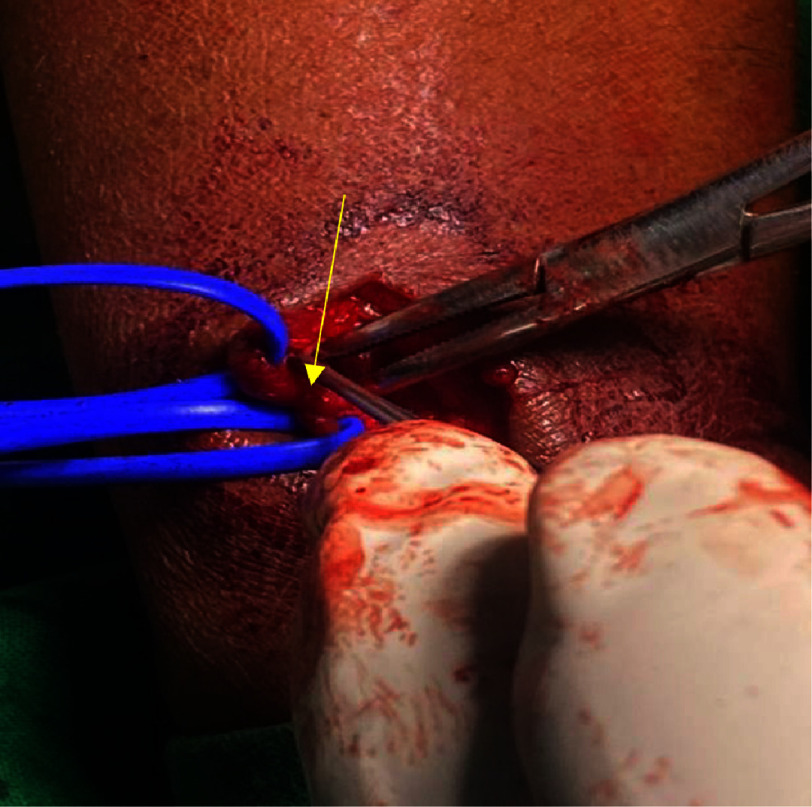
Intra-operative image of the AV fistula (yellow pointer).

**Figure 5.**
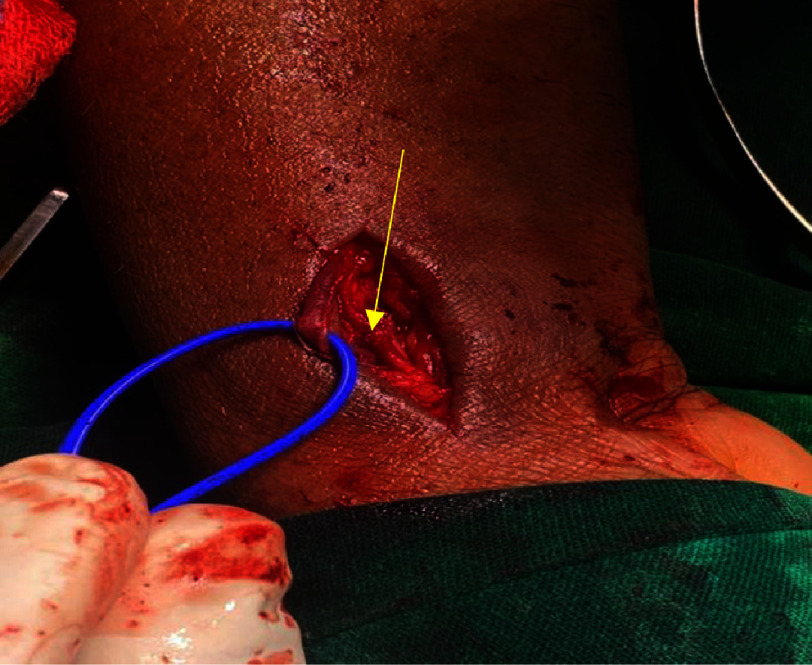
Post-AV fistula ligation (yellow arrow, small blue suture seen at the site of ligation).

**Figure 6.**
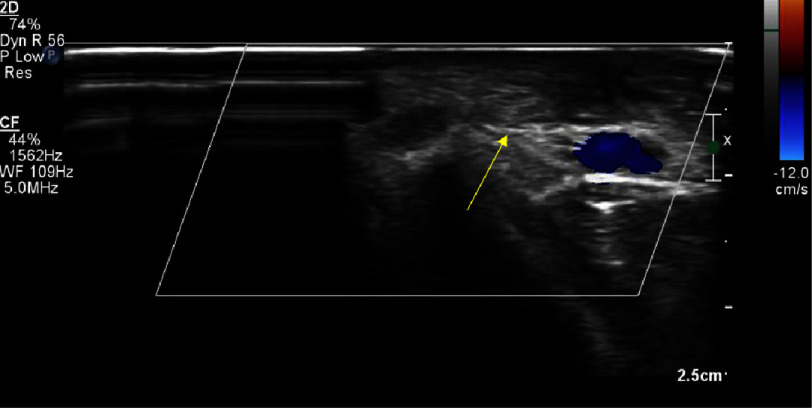
Follow-up arterial Doppler showing a thrombosed AV fistula (yellow arrow).

**Figure 7.**
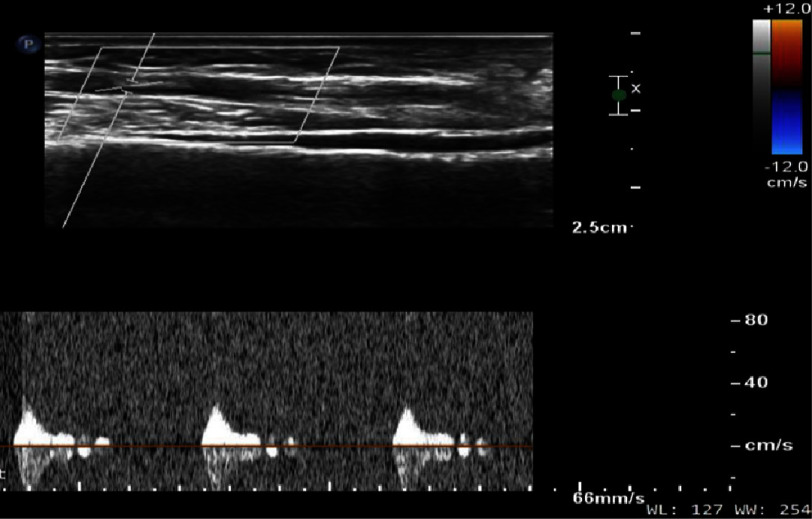
Arterial Doppler on follow-up showing absence of flow gradient between the radial artery and cephalic vein.

Successful right radial AV fistula ligation was performed under local anesthesia (Figures 4, 5). On subsequent follow-up visit after four weeks, the patient reported a reduction in the frequency and intensity of the local symptoms and improved sleep; thrill was not palpable. Arterial Doppler analysis of the right radial access site revealed that the fistula had thrombosed and there was no flow gradient between the radial artery and cephalic vein (Figures 6, 7). At one year of follow-up, the patient remained asymptomatic, with adequate functioning of the affected hand, and Doppler showed no flow across the site of the original fistula.

## Discussion

Transradial access is often preferred over femoral access because of reduced blood loss, improved early mobilization, better patient acceptability, and fewer vascular complications^1–3^. However, studies have shown that transradial access also has major and minor access-related complications^6^. With the increasing use of this access route, there is a necessity for awareness, early identification, and prevention of these complications^3,6^. Radial access complications can be classified as intra-procedural and post-procedural (Table 1). Intra-procedural complications can be associated with bleeding (radial artery perforation) or without bleeding (radial artery spasm or dissection). The postprocedural complications associated with bleeding are forearm hematoma and compartment syndrome, and those not associated with bleeding include radial artery occlusion, pseudoaneurysm, AVFs, nerve damage/regional pain syndrome, and local site infections^6^.

**Table 1:** Complications of transradial access for coronary procedures [adapted from [6]].

Intraprocedural		Post- procedural	
Complications associated with bleeding	**Complications not associated with bleeding**	**Complications associated with bleeding**	**Complications not associated with bleeding**
Radial artery perforation	Radial artery Spasm • Traumatic eversion • Catheter entrapment	Forearm hematoma and compartment syndrome	Radial artery occlusion • Symptomatic • Asymptomatic
	Arterial dissection		Pseudoaneurysm
	Catheter kink		AV Fistula
			Nerve damage/ Regional pain syndrome
			Infection

Radial AVF is very rare, and some studies and trials, such as the RIVAL trial, have shown an incidence of less than 0.03−0.08%^3,6,7^. However, with the increasing use of radial access, more cases of radial AVF have been reported^2,4^. When a vein, such as the cephalic vein, is near the radial artery puncture site, there is a risk of AVF. In these situations, the needle used to puncture the radial artery for arterial access can inadvertently puncture the nearby venous tributary, resulting in an unnoticed combined arterial and venous puncture.

In most cases, this communication seals; however, if it fails to seal, an AV fistula results^2^. In a prospective study by Kelm et al. in patients undergoing cardiac catherization via the femoral access, female gender, systemic hypertension, intra-procedural higher heparin dose were associated with an increased risk of femoral AVF formation, however the number of sheaths used at the arterial site did not affect the risk^8^.

A similar association can be extrapolated for transradial access due to the similar mechanism of AVF formation at both access sites^2,8^. Most often these radial AV fistulas manifest with signs of venous dilatation, swelling and a palpable thrill, rarely high output heart failure and steal syndrome with neurological manifestations can result^2,4,7,9^. A persistent AV fistula can, in the long run, result in high-output cardiac failure, particularly in those with previously compromised cardiac function^10^. This occurs due to increased venous return as a result of increased blood flow via the fistula from the high-resistance artery to the lower-resistance vein^2,10^.

**Figure 8.**
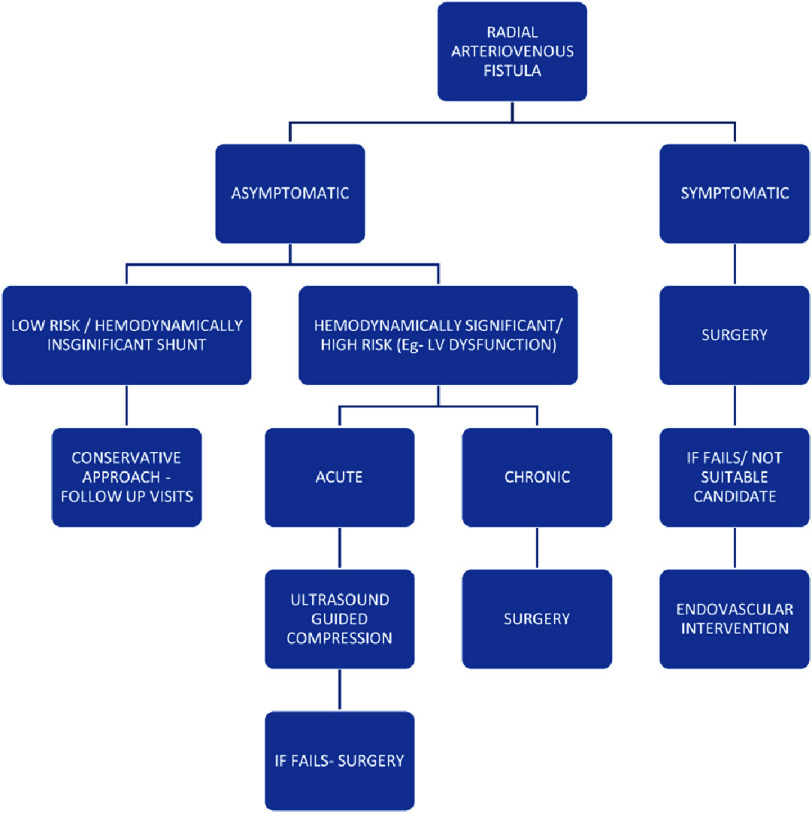
Algorithm for the management of iatrogenic radial arteriovenous fistula.

Possible strategies for management of the radial AV fistulae include conservative approach with follow up in asymptomatic or those with hemodynamically insignificant shunts^7,9,11^, non-invasive prolonged compression with a hemostatic band, and invasive approaches like stenting with covered stents, and surgical ligation, in those who are symptomatic (Figure 8)^7,11^.

The radial artery can be easily compressed because of its superficial location and small lumen. However, studies have shown that this compression was effective in treating mainly early stage fistulas (within about a month) and was less successful in fistulae detected late (approximately a year). If compression fails, endovascular treatment or surgical interventions can be attempted^11^. Surgical approaches include ligation (for easily identified fistulae), excision, or repair of the radial artery (in mixed/poorly discernible fistulae, where there is considerable radial artery damage). Endovascular treatment involves balloon-assisted percutaneous embolization; however, it is less preferred because of the small artery size and embolism risk^11^. In the cases reported by Dehghani et al.^2^ and Moorthy et al.^9^, patients were conservatively managed and remained asymptomatic at follow-up. In a case series by Okam et al.^4^, surgical management was preferred due to persistent symptoms.

In the case reported here, because there was no improvement after conservative management and considering the patient’s cardiac history, ejection fraction, and distressing symptoms, surgical ligation of the fistula was performed. We are not sure if the reported increase in pulmonary artery pressure in the two echocardiograms performed during the fistula evaluation was due to the fistula or a result of a more detailed assessment in the second ECHO. Invasive pulmonary pressure was not measured. There is no clear preferred treatment modality for radial fistulas. In most reported cases, if the fistulas were symptomatic, surgical management was performed, either as an initial approach or after a failed trial of compression, as was done in our case.

In our case, the same radial artery was used on two separate days for access during CAG and PTCA. No imaging was used to guide the radial-access puncture for either procedure. Despite ultrasound-guided punctures being recommended for both radial and femoral access, their use remains limited in resource-limited high-volume settings. This can result in an increased risk of vascular complications, such as AVF. Furthermore, the same radial access site used for two separate staged procedures could have increased the risk of AVF in the patient. Some possible strategies to prevent AV fistula include reducing the number of times an artery is accessed, using sheaths smaller than the arterial diameter, and imaging guidance for punctures^2^. While other case reports suggest that improved operator experience can reduce complications, in our case, the radial punctures in both cases were performed by experienced operators on the team^2^.

## Conclusion

Radial access for coronary interventions is also associated with vascular complications, albeit less frequently than femoral access. Since radial access is commonly used nowadays, awareness of the presentation and risk factors of these complications can enable their prevention, early detection, and appropriate management. Imaging guidance should be used to obtain vascular access punctures to reduce the risk of complications.

## What have we learnt?

Radial access can also be associated with complications, including arteriovenous fistulas. Even experienced operators may inadvertently experience these complications. Hence, knowledge of the complications, possible management options, and prevention strategies for radial access complications should be known to all operators performing coronary interventions. When available, imaging guidance should be used.
